# Food-Grade PE Recycling: Effect of Nanoclays on the Decontamination Efficacy

**DOI:** 10.3390/polym12040822

**Published:** 2020-04-04

**Authors:** Yannick Laridon, François Touchaleaume, Nathalie Gontard, Stéphane Peyron

**Affiliations:** UMR IATE, CIRAD, INRAE, Montpellier SupAgro, Université de Montpellier, 2 Place Pierre Viala, 34060 Montpellier, France; francoistouchaleaume@gmail.com (F.T.); nathalie.gontard@inrae.fr (N.G.); stephane.peyron@umontpellier.fr (S.P.)

**Keywords:** decontamination, recycling, nanoparticles, food safety, food packaging

## Abstract

Although PE-based nanocomposites are gaining interest within the food packaging industry for their outstanding functional properties, their end-of-life has been poorly studied. The lack of identification of such materials suggests that they could end-up in the recycling pathway optimized for the decontamination of un-filled PE. The objective of the present work is to understand and quantify the mechanisms involved in the high temperature desorption of surrogates for PE nanocomposites filled with organo-modified montmorillonite (PNC), compared to conventional PE. An original experimental setup was coupled with a modelling approach to identify the two phenomena involved in the decontamination process: diffusion of the surrogate into the bulk and its evaporation at the surface. A sweep of experimental temperatures enabled the determination of diffusion and evaporation parameters for PE and PNC and the activation energies related to the diffusivity among those two materials. The effects of the introduction of clay nanofillers onto the decontamination process have been explained and recommendations for the recycling pathway have been put forward.

## 1. Introduction

Packaging, which is used to prevent food spoilage from production to consumption, is a key point for globalization, harmonization and trade barriers in terms of consumer safety and waste management. The development strategy of food packaging nanocomposites (PNC) aims (1) to enhance their mechanical and barrier properties, with a very special challenge for eco-friendly bio-plastics for environmental purposes (nano-bio-composites) and/or (2) to endow them with innovative functionalities (antimicrobial, gas perm-selectivity, nano-bio-sensors, etc.) for an active and intelligent packaging development. The market of nanotechnologies is dominated by the food packaging sector which amounted to over 20% of the total nanotechnology market in 2015, grossing €700 billion by 2020 with 2/3rd market share for food packaging.

However, the wide-scale use of engineered nanocomposite materials for food packaging [1] raises important questions about environmental and safety issues that could strongly hinder the nanotechnology market development. Indeed, the introduction of nanoparticles in such new composites materials presents particular challenges, especially with regard to safety issues, and should induce new technical constraints on the mechanical recycling process initially developed for neat polymers. The European Union clearly sees mechanical recycling as the favorite route for the management of plastic waste [2] and even if chemical recycling is starting to show promising advances [3] physical routes are still prevailing. While public health authorities push forward the safety evaluation of recycled post-consumer packaging, the introduction of nanoparticles in such new composite materials presents particular challenges, especially with regard to safety issues, and should induce new technical constraint on the mechanical recycling process initially developed for neat polymers. Up to now, recycling post-consumer plastics into food contact materials (FCM) mainly focused on PET [4] and, as any other FCM, in Europe at least, recycled PET must comply with the CE/10/2011 [5] European regulation. In addition to inertia criteria, recycled materials are placed on the market on the condition that they are obtained by a mechanical recycling process authorized by EU regulation CE/282/2008 [6].

Consumer safety is ensured by assessing the chemical decontamination efficiency resulting from approved recycling technology. The main task of this key step of the recycling process is to remove traces of chemical contaminants from the materials. Technically, it includes a material deep-cleansing operation to reduce contaminant levels by prior incubation at high temperature and by an inert gas/vacuum treatment [7]. The procedure evaluation submitted by the EFSA is based on especially designed challenge tests including the measure of the post-processing residual concentration of sets of surrogate contaminants [8]. This approach rests upon a comparative analysis between the measured residual concentration (Cres) and a calculated concentration (Cmod), which corresponds to the expected migration of the substances calculated using recognized conservative migration models, the final concentration in food being defined by the human exposure threshold value for genotoxic chemicals. This approach proved to be adapted to the specific case of PET recycling technologies, but does not seem to be reliable for polyolefins which exhibit poor barrier properties and reduced thermal stability [9]. As a consequence, no standard recommendations of thermal desorption technologies are currently available for the decontamination of polyethylene or polyolefin blends and recycling processes were not considered of concern by EFSA if the material came from closed- and controlled-loop recycling (regulation CE/282/2008 [6]). The production of nanocomposite material is an alternative way to increase the stability of recycled plastic, but the further recyclability of these composite materials has not so far been assessed. Up to now, there is very little literature data available concerning the transport mechanisms of organic contaminants in nano-composite materials at high temperature as applied during recycling processes. This issue is of the utmost importance for the apprehension of nanocomposite recyclability control.

Previous investigations which focused on the mass transport properties of food packaging demonstrated that nano-sized fillers incorporation induces a decrease in the diffusivity of low molecular weight molecules [10]. This statement appears to be beneficial in terms of the contact of nanocomposite packaging with food due to the limitation of the migration of plastic additives, but could appear to be detrimental in terms of thermal decontamination due to the reduction in the desorption of contaminants. Moreover, a historical role played by nano-sized clay platelets could have some effect on the decontamination step of the plastic recycling process. Indeed, great scientific efforts have been made since the 1980s on the subject of smectic clays which have started to be considered as efficient materials to remove organic contaminants from the environment [11]. The best way to improve this natural capacity to adsorb organic contaminants consists of modifying the clays with quaternary ammonium cations, which not only improves the available specific surface area but also the organophylicity of the mineral. Later, those two phenomena also became advantageous for the elaboration of clay-polymer nanocomposites and most current clay nanofillers contain quaternary ammonium surfactants [12]. To our knowledge, the adsorption capacity of such clays has not been assessed when considering the purification of contaminated nanocomposites that contain this nanofiller.

The objective of this study is to push forward the understanding of the desorption mechanisms of contaminants of a standard polyethylene-based nanocomposite material at high temperature. The biphenyl, a low molecular weight molecule that belongs to the polychlorinated biphenyls (PCB) family, is a major food contaminant, and structurally close to the phenylcyclohexane selected in the list of contaminants recommended by the EFSA [8]; it was selected as a model surrogate to describe the impact of nanoclays (organo-modified montmorillonites) on the different transfer parameters which describe the desorption of the molecule at different temperatures.

## 2. Materials and Methods

### 2.1. Materials

High-purity surrogates (biphenyl, benzophenone, methyl stearate and octabenzone) were provided by Sigma-Aldrich (Saint-Louis, MO, USA). Linear low density polyethylene (grade LL 1002YB, melt flow index of 2.0 g/10 min, density of 0.918 g·cm^−3^), supplied by Exxon Mobil Chemical (Irving, TX, USA), was chosen as the polymer matrix (labelled “PE” in the present work). Clay was provided as a 40%-loaded masterbatch also based on linear low density polyethylene. The organically modified montmorillonite was Nanomer I44P (containing 38% dimethyl dialkyl (C_14-18_) Ammonium as checked by thermogravimetric analyses) from Nanocor (Hoffman Estates, IL, USA). The mineral content of the masterbatch was measured at 23.5% using TGA.

### 2.2. Virgin Material Processing

Two preparation routes were chosen to cover the two usual sample shapes used in polymer recycling: pellets and film strips (chosen to mimic the common polymer flakes). The decontamination was monitored using TGA for the pellets while, for the films, spiked samples were displayed in a ventilated oven and periodical samplings allowed the quantification of the decontamination process.

The PE and clay masterbatches were extruded in a Eurolab 16 twin-screw extruder (Thermo Fisher Scientific, Waltham, MA, USA) with an L/D ratio of 40, a 16 mm screw diameter, running at 600 rpm with a 1 kg·h^−1^ total throughput and heated from 160 °C to 180 °C from hopper to die. The materials were then water-cooled and pelletized with rotative knives.

Film samples were obtained by adapting a 150 mm-wide flat die thermally set at 180 °C instead of the annular die and calendaring between 70 °C-set rolls.

Similar processing steps were applied to pure PE, both for the production of pellets and film. The virgin processed material was labelled PE and the polymer nanocomposite PNC. The final mineral content of the PNC (both in film and pellet shapes) was checked using TGA at 5.9 w% (±0.4%). The average heights and radius of the pellets were respectively 3.19 (±0.13) and 1.51 (±0.06) mm for PE, 2.94 (±0.09) and 1.68 (±0.07) mm for the PNC. The average film thicknesses were 190 (±20) and 161 (±23) μm for PE and PNC, respectively.

### 2.3. Characterization of Clay Dispersion

X-ray diffraction (XRD) is a common analytical tool used to representatively quantify the clay dispersion state of polymer nanocomposites. XRD experiments were performed using an “in-house” setup detailed hereafter: a high brightness low power X-ray tube, coupled with aspheric multilayer optic (GeniX3D from Xenocs, Grenoble, France) delivered an ultralow divergent beam (0.5 mrad; flux: 20 MPhotons·s^−1^; λ=1.5418 Å). A transmission configuration was used and the scattered intensity determined by a 2D pixel “Pilatus” detector at a distance of 20 cm from the sample, which consisted of five stacked film thicknesses. The obtained intensities were corrected by transmission and the empty cell contribution was subtracted.

### 2.4. Spiking

Prior to the spiking step, the virgin samples were dried for 24 h at 60 °C in a vacuum oven and kept in a desiccator for one week over P_2_O_5_. Then, 5 g of pellets were mixed with 20 mg of surrogate, the bottles were sealed, and stored at 40 °C under rotary agitation.

Five grams of film strips were displayed in sealed flasks containing 75 mL of dichloromethane and 1 g of surrogate and kept under stirring at room temperature.

In addition to biphenyl, three other surrogates were used for the spiking of the film strips: benzophenone, methyl stearate, and octabenzone, to cover a wider range of molecular weights and vapor pressures.

### 2.5. Decontamination Tests

After 2 weeks, 16 spiked pellets were removed from the bottle, rinsed with distilled water and wiped off. Eight pellets were then displayed on the measuring pan of the TGA for decontamination monitoring. Experimental temperatures were chosen from the following set: 55 °C, 70 °C, 85 °C, and 100 °C. The remaining pellets were subjected to pure dichloromethane for extraction to obtain the initial concentration ($c_{0}$).

**Figure 1:**
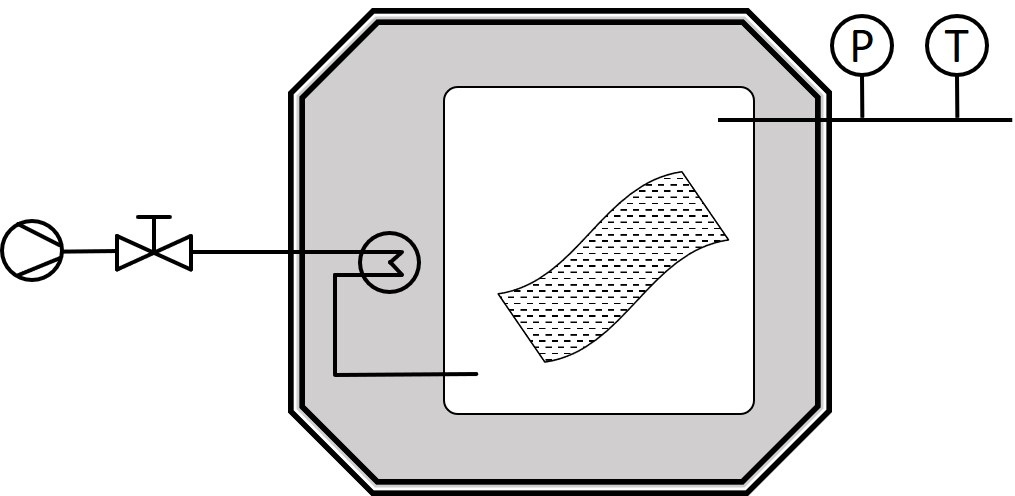
Experimental device employed for the decontamination of film strips.

After 5 spiking days, the film strips were removed from the flasks, wiped off, and placed in a vented cell kept in an oven (Figure 1). Samples were periodically removed and subjected to dichloromethane for extraction in order to quantify the surrogate remaining in the film.

### 2.6. Characterization

Regardless of the analytical process employed (whether thermogravimetric monitoring completed by two GC quantifications, or periodical GC quantifications of materials displayed in the vented cell), each experiment was repeated at least twice.

#### 2.6.1. Thermogravimetric Analyses

Decontamination was evaluated using TGA (TGA2, Mettler Toledo) and by heating 8 spiked pellets (that weighed around 150 mg) under nitrogen atmosphere (flow rate of 50 mL·min^−1^). The heating profile included a stabilization time of 1 min at room temperature followed by a quick heating ramp (30 °C·min^−1^) from room temperature to the chosen decontamination temperature. The pellets were then kept at this temperature and the total weight was monitored during the simulated decontamination process. The quantification of the pellet contamination level was carried out using gas chromatography before the thermal treatment (for similarly prepared pellets) and after the TGA analyses (for the thermally decontaminated pellets). Blank tests were also run on empty pans in order to quantify the buoyancy phenomena induced by quick temperature changes that occur in the oven.

#### 2.6.2. Gas Chromatography

The concentration of additives in the dichloromethane was determined using an Agilent 7890A gas chromatograph (Santa-Clara, CA, USA) equipped with a 7693A Automatic Sampler and a flame ionization detector. Data were collected and processed using the ChemStation OpenLab Software (rev. B.04.03, Santa-Clara, CA, USA). An HP-5 (5%-phenyl) methylpolysiloxane capillary column of 32 mm ID, 30 m length, and 0.25 mm film thickness was used. The temperature program for the gas chromatography was as follows: the initial oven temperature was 35 °C, held for 5 min, followed by heating with a linear gradient of 6 °C·min^−1^ to 270 °C, and held for 15 min. The injector temperature was 260 °C, and injection was performed with the split ratio of 40:1. The external calibration method was performed in a range of 0.1–200 μg·mL^−1^ with six repetitions for each concentration to obtain the calibration equation.

## 3. Diffusion Modeling: Theoretical Background

### 3.1. Description of the Systems

#### 3.1.1. Hypotheses

The diffusion into the bulk is assumed to be homogeneous and isotropic. In the case where nanoparticles were dispersed in the PE matrix, the size and distribution of the nanoparticles was not modeled. We chose to remain at a macroscopic level to describe the system, which allowed us to maintain the homogeneous and isotropic hypotheses. The diffusion coefficient is assumed to be constant over time, independent of the concentration of contaminant in the bulk (according to a Fickian diffusion model). The samples used consisted of pellets or films. The pellets are considered as cylinders of radius *R* and height *H*. As for the films, their thickness (from 157 to 196 μm) was noticeably lower than their other dimensions (a few cm), which is what we considered to be the case in the infinite plane sheet case.

#### 3.1.2. Diffusion in the Materials

The diffusion is modelled using Fick’s second law:

(1) $$\frac{\partial c}{\partial t}=D\Delta c$$

where *c* is the contaminant concentration in the domain and *D* the diffusion coefficient. The initial contaminant concentration, $c_{0}$, is assumed to be uniform within the domain.

#### 3.1.3. Boundary Conditions

The pellets were placed in a well-agitated atmosphere, typically with N_2_, with infinite volume, so that the exterior contaminant concentration was permanently null. A flux boundary condition was applied at the boundary, following Fick’s first law:

(2) $$D\nabla c=-h_{m}\left(c_{\Gamma }-c_{ext}\right).$$

Here, $h_{m}$ is the external mass transfer coefficient, $c_{\Gamma }$ the concentration at the boundary, and $c_{ext}$ the concentration in the atmosphere surrounding the samples.

In order to determine the mass transfer coefficient and diffusivity, two cases were considered: a diffusion-limiting case (the most common case) and a case that included evaporation. For the former, the simplest analytical solutions were used. The latter was parametrized using the Biot number for mass transfer, defined as:

(3) $$Bi=\frac{L_{c}h_{m}}{D}$$

where $L_{c}$ is a length characteristic of the geometry used: in the case of pellets, the radius, and in the case of films, half of the thickness (for symmetry reasons). The two extreme cases, Bi≫1 and Bi≪1, indicate the diffusion-limiting case (common case) and the evaporation-limiting case, respectively.

### 3.2. Analytical Solution

#### 3.2.1. Film Geometry

The fractional quantity of the contaminant, derived from Equation (1) for the plane sheet, is given by Equation (4).53 from Crank 1975 [13]:

(4) $$q_{z}\left(t\right)=\int_{0}^{H}\frac{c(t,z)}{c_{0}}dz=2\sum_{n=1}^{\infty }\frac{Bi^{2}}{\alpha_{n}^{2}\left(\alpha_{n}^{2}+Bi^{2}+Bi\right)\exp-4\frac{\alpha_{n}^{2}D}{H^{2}}t},$$

where *H* is the film thickness and the $\alpha_{n}$ are the positive roots of:

(5) αtanα=Bi.

For the diffusion-limiting case, the simpler Equation (4).18 from Crank 1975 [13] was used.

#### 3.2.2. Pellet Geometry

Similarly to the previous section, the radial part of the fractional amount of contaminant is written as follows (Equation 5.49 from Crank 1975 [13]):

(6) $$q_{r}\left(t\right)=\int_{0}^{H}\frac{c(t,r)}{c_{0}}dr=4\sum_{n=1}^{\infty }\frac{Bi^{2}}{\beta_{n}^{2}\left(\beta_{n}^{2}+Bi^{2}\right)\exp-\frac{\beta_{n}^{2}D}{R^{2}}t}$$

where *R* is the pellet radius and the $\beta_{n}$ are the positive roots of:

(7) $$\beta J_{1}\left(\beta \right)-BiJ_{0}\left(\beta \right)=0.$$

For the diffusion-limiting case, the simpler Equation (5).23 from Crank 1975 [13] was used. Finally, using the variable separation method with Equations (4) and (6), the fractional amount of contaminant in the sample (pellet) can be written:

(8) $$q\left(t\right)=q_{r}\left(t\right)q_{z}\left(t\right)$$

### 3.3. Estimation of Parameters

The diffusion coefficient *D* and external mass transfer coefficient $h_{m}$ (used to calculate the Biot number, Equation (3)) were the two variables of interest. They were determined by fitting Equations (4) and (8) to experimental data, for the films and the pellets respectively, acquired as described in Section 2.5.

For the films, the GC analysis directly provides the fractional amount of contaminant in the samples over time.

In the case of pellet samples, Equation (8) gives the fractional variation of contaminant mass, but the TGA gives the total sample mass over time. To compare the experimental measure to the simulation, Equation (8) was used to express the simulated total sample mass over time. The total mass $m_{T}$ measured using TGA can be written as the sum of $m_{M}$, the material mass, assumed to be constant over time and $m_{C}$ the mass of embedded contaminant. The latter can be expressed as:

(9) $$m_{c}\left(t\right)=q\left(t\right)m_{c}\left(t=0\right)=q\left(t\right)c_{0}m_{M}$$

At t=0, we then have:

(10) $$m_{T}\left(0\right)=m_{M}+c_{0}m_{M}=m_{M}\left(1+c_{0}\right)$$

Combining the previous equations leads to the following expression for the total pellet mass over time:

(11) $$m_{T}\left(t\right)=m_{T}\left(0\right)\frac{1+c_{0}q\left(t\right)}{1+c_{0}}$$

### 3.4. Estimation of Uncertainty

The experimental variance for the estimation of *D* and $h_{m}$ was computed as:

(12) $$V_{exp}=e_{r}^{2}+e_{a}^{2}+RMSE^{2}$$

where $e_{r}$ and $e_{a}$ are the reproducibility and accuracy of the TGA apparatus and RMSE the root mean square error of the fit. This variance was propagated in the determination method to better estimate the effect of the determination method:

(13) $$V={\left(J^{T}J\right)}^{-1}V_{exp}$$

where *J* is the Jacobian matrix of the analytical solution (Equation (11)).

Moreover, one major source of error was the uncertainty on the initial contaminant concentration $c_{0}$ in the matrix. We therefore did two additional fittings using the confidence intervals on $c_{0}$ to estimate the propagated standard error $\sigma_{c_{0}}$ on the estimated parameters, so that the overall uncertainty can be expressed as:

(14) $$\sigma =\sqrt{\sigma_{c_{0}}+V}$$

### 3.5. Implementation

Equations were implemented numerically using Matlab (The MathWorks, Naticks, MA, USA). The simulation results using these equations were fitted to the experimental data following the least-squares method in minimizing the root mean square error (RMSE).

As stated previously, two cases were considered: the diffusion-limiting case, for which the Biot number was set at a high value (typically 10^4^–10^5^) and only *D* was fitted; and one where both *D* and $h_{m}$ were fitted, to investigate the competition between diffusion in the domain and surface evaporation.

## 4. Results and Discussion

### 4.1. Microstructure of the Polymer Nanocomposite

**Figure 2:**
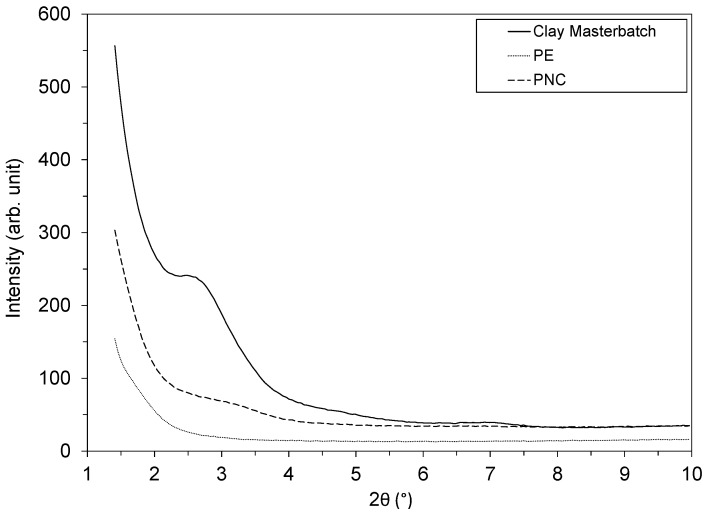
X-ray diffraction patterns of PE, PNC and Clay Masterbatch.

The dispersion state of the clay platelets among the polymer matrix directly impacts the macroscopic performances of the nanocomposites [14], including the transfer properties [15]. The XRD diffractogram of the pure PE matrix did not reveal any diffraction peak, as expected and revealed in Figure 2. However, for the clay masterbatch, a diffraction peak was observed at 2θ=2.5°, corresponding to a clay interlayer distance $d_{001}=3.6$ nm. This value is higher than the 1.3 nm interlayer spacing reported for the modified clay selected for the present masterbatch preparation [16], thus indicating that the masterbatch exhibited a primary intercalation state. After dispersion of the masterbatch into the PE matrix, this peak strongly reduced in intensity and appeared as a broad signal between 2 and 3.5°. Regarding the microstructure, this signal modification reflected a loss of crystalline order, revealing an improved intercalation level and the presence of exfoliated platelets. The presently prepared PNC exhibited a typical clay dispersion state leading to improved mechanical and barrier properties in comparison with pure PE. Further experiments enabled quantifying the effect of the clays onto the decontamination process.

### 4.2. Surface Evaporation

**Figure 3:**
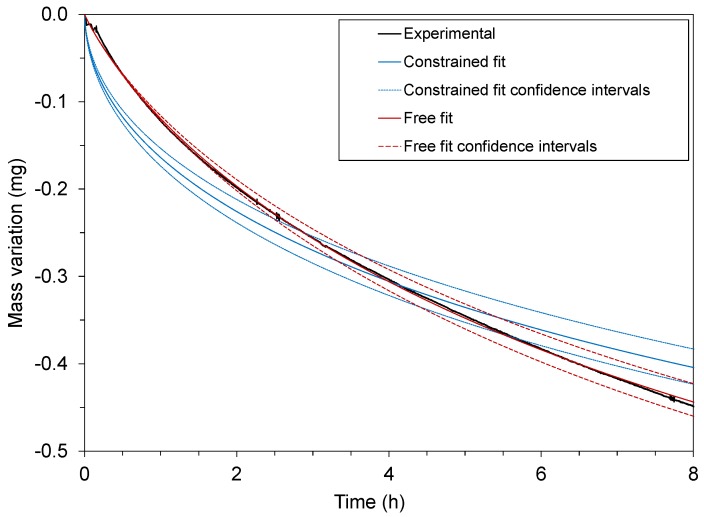
Comparison of the free fit (surface evaporation and domain diffusion compete) and the constrained fit (diffusion-limiting) cases with the experimental desorption data acquired with PE pellets contaminated with biphenyl, at 70 °C.

After thermally decontaminating spiked pellets and gravimetrically monitoring the phenomenon (Figure 3), two diffusion cases were considered and the corresponding models were applied to the experimental data in order to determine the diffusion parameters. Figure 3 reports that the parameter determination in the diffusion-limiting case (constrained fit) yielded poor fits (as exemplified in Figure 3 for the pellet geometry) compared to the case where the two phenomena, i.e., diffusion in the bulk and surface evaporation, compete (free fit). In the example shown in Figure 3, the free fit yielded an RMSE of 2.3 μg, whereas the constrained fit yielded an RMSE of 28.9 μg. A similar comparison could be made for all the data acquired, with RMSE consistently one order of magnitude higher for the constrained fit than for the free fit. In addition to the RMSE, Figure 3 clearly shows that desorption kinetics is better for the free fit.

This underlines the fact that, in this case (the decontamination of pellets at high temperature), the surface evaporation of the contaminant can be a limiting phenomenon and should therefore be taken into account.

The vapor pressures (*P*) were calculated according to the modified MacKay method [17], expressed as:

(15) $$\ln P=K_{f}\left(4.4+\ln T_{b}\right)\left(1.8\frac{T_{b}-T}{T}-0.8\ln\frac{T_{b}}{T}\right),$$

where $T_{b}$ and *T* are the boiling point and the experimental temperature, respectively; $K_{f}$ is a structural factor of Fishtine which corrects many polar interactions [18]. It has a value of 1 for non-polar and monopolar compounds, a value of 1.04 for compounds with a weak bipolar character, a value of 1.1 for primary amines and a value of 1.3 for aliphatic alcohols.

Experimental data were obtained with several contaminants for samples with a film geometry (see Section 2.2). The data underlined a clear correlation between the vapor pressure *P* of the contaminants and the external mass transfer coefficient $h_{m}$.

The lower the vapor pressure, the weaker the external mass transfer phenomenon, as non-volatile compounds (i.e., with low vapor pressure) rarely evaporate from the material/air interface and hence favor evaporation-limiting cases.

**Figure 4:**
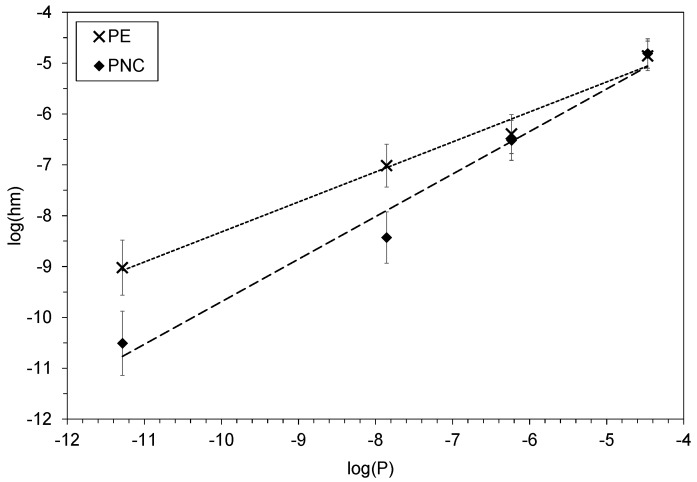
Relationship between the external mass transfer coefficient (Equation (2)) and the theoretical vapor pressure (Equation (15)) of the contaminants. Dotted lines are for readability.

Moreover, a clear effect of the presence of nanoparticles in the matrix was noted on the ability of the same contaminants to evaporate (Figure 4): the PNC material presented consistently lower external mass transfer coefficients, indicating that, for a given compound, evaporation processes were more limiting for the PNC than for PE. This could be linked to more favorable interactions between the compound with a PNC surface than that with a PE surface. It should be noted that the fitted external mass transfer coefficient $h_{m}$ does not only account for surface evaporation, but also encompasses the various phenomena that can occur at the surface of the material, which acts as an obstacle to decontamination.

### 4.3. Effect of Temperature and Activation Energy Estimation

**Figure 5:**
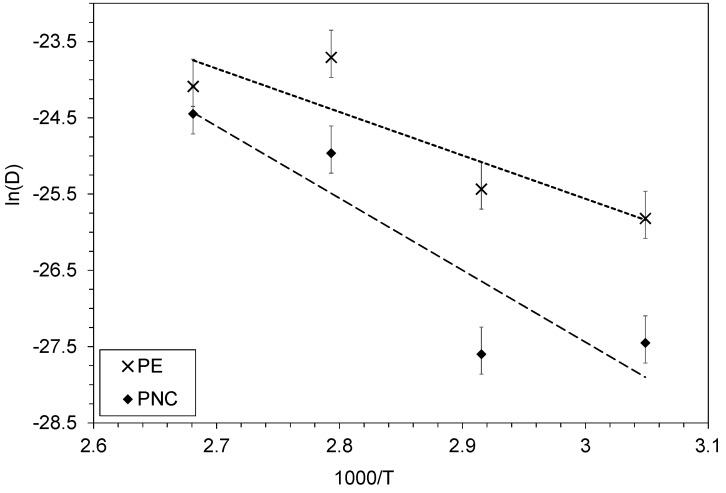
Arrhenius plot for biphenyl in PE and PNC. Crosses and diamonds stand for experimental data with the associated error bars while the dash lines represent the modeled curves obtained from the former.

The diffusivity of biphenyl in both materials was determined at four temperatures, which enabled the estimation of the activation energy for both cases (Figure 5).

Activation energy $E_{a}$ was estimated by fitting the experimental data to an Arrhenius relationship:

(16) $$D\left(T\right)=D_{0}\exp-\frac{E_{a}}{RT},$$

where $D_{0}$ is a pre-exponential factor.

This yielded activation energies of 47.3 and 78.5 kJ·mol^−1^ for PE and PNC materials, respectively. This is in agreement with the literature, both regarding the order of magnitude and the fact that they are positive, describing the case where diffusivity is higher for high temperatures.

In practical terms, it can be more convenient to speak in terms of characteristic times to have an idea of the time taken by the contaminant to leave the polymer bulk. Classically, characteristic diffusion time is expressed as:

(17) $$t_{c}^{D}=\frac{L_{c}^{2}}{D}.$$

Similarly, characteristic time for surface evaporation can be written:

(18) $$t_{c}^{h}=\frac{L_{c}}{h_{m}}.$$

The Biot number can thus also be expressed as:

(19) $$Bi=\frac{t_{c}^{D}}{t_{c}^{h}}.$$

**Figure 6:**
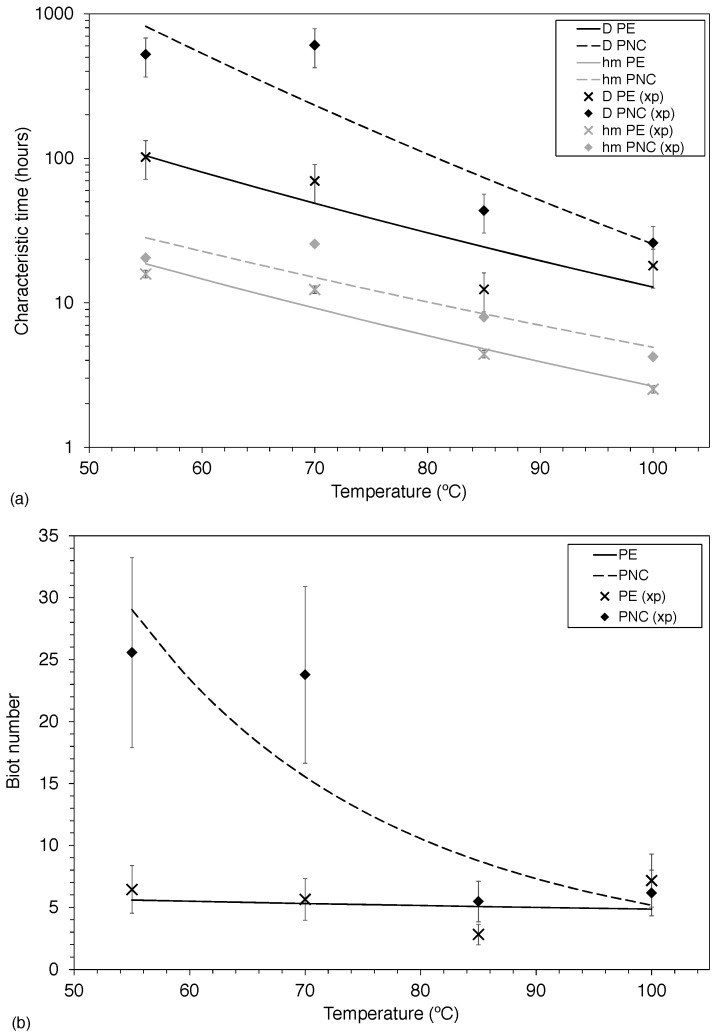
Effect of temperature on (**a**) characteristic times (Equations (17) and (18)) and (**b**) Biot number (Equation (19)). Experimental data are denoted by (xp).

Figure 6 gives the characteristic times for the two phenomena (diffusion and surface evaporation) involved in polymer decontamination, as a function of temperature (Figure 6a), as well as the Biot number for corresponding temperatures (Figure 6b) that is to say the ratio of the characteristic times (Equation (19)). The characteristic times shown correspond to the times for which 99.7 and 99.9% of the materials were decontaminated, for PE and PNC (resp.). The substantial error bars reported on Biot number at the lower temperatures for PNC can be explained by the heterogeneity of the nanocomposite samples, which may be higher than for virgin LDPE.

Consistently with what was previously discussed, the inclusion of nanoparticles negatively impacts the decontamination, in terms of diffusion or surface evaporation. For the first, diffusion characteristic time is twice as long for PNC than for PE at 100 °C (and nearly eight times at 55 °C), for the latter, the ratio stays relatively the same, the evaporation characteristic time being 1.9 times higher for PNC than for PE at 100 °C (compared to 1.5 times at 55 °C). As in both materials diffusion is the limiting factor, the characteristic diffusion time is representative of the time it takes to fully (or near-fully) decontaminate both materials of biphenyl. The two materials exhibit relatively different behaviors regarding the two phenomena involved: while, for PE, diffusion is the limiting factor, being consistently around five times slower than surface evaporation, for PNC, the Biot number only reaches the same value as for PE at high temperatures (over 95 °C). The extreme case is at 55 °C, where the diffusion is nearly 30 times slower than surface evaporation.

By applying the same experimental protocol to contaminants with various molecular weights, it may be possible to infer an empiric law (similar to the Piringer model [19]) that would give an estimate of the decontamination time for any given contaminant in PE and PNC, provided that we can have its molecular weight (for diffusion prediction) and vapor pressure (for surface evaporation prediction), and determine the optimal temperature for the decontamination process.

### 4.4. Tortuosity and Effect of Nanoparticles

Tortuosity is defined as the ratio between the diffusivity in virgin polymer (PE) and the diffusivity in the composite material (PNC):

(20) $$\tau =\frac{D_{PE}}{D_{PNC}}.$$

Usually, tortuosity is a geometric factor that represents the increase in the path length for the diffusing substance, typically caused by the presence of nanoparticles in an intercalated-exfoliated structure as confirmed by XRD for the present PNC. Similarly to the external mass transfer coefficient, Equation (20) expresses *apparent* tortuosity that can include the effects of *true* geometric tortuosity, but also accounts for the changes in bulk diffusivity due to the presence of nanoparticles, a phenomenon which occurs at the matrix–nanoparticle interphase.

**Figure 7:**
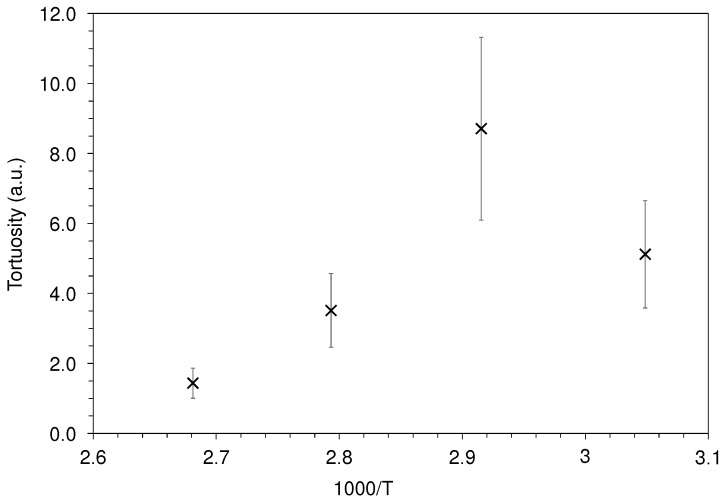
Tortuosity values as a function of temperature.

Figure 7 highlights the effect of nanoparticles on the decontamination of PE. The substantial error bars at the lower temperatures were mainly attributed to the significant uncertainty previously identified for PNC values. Regardless of the experimental temperature, the tortuosity is higher than 1, revealing a reduced diffusivity of biphenyl molecules within the PNC in comparison with PE. This can be attributed to the longer pathway encountered by the surrogates diffusing out of the nanocomposites compared to the easy-to-cross pure PE material as widely reported for filled polymer systems [20]. This pure geometric consideration should not be influenced by temperature, considering that, even if biphenyl mobility increases with temperature, this would similarly impact its bulk diffusion among the two materials. The present results indicate that tortuosity globally diminishes with increasing temperature. Surprisingly, the experiments held at 55 °C (corresponding to 1000/T=3.05 K^−1^) lead to a peculiar result, tortuosity being lower than for T=70 °C (corresponding to 1000/T=2.92 K^−1^). This may be linked to the physical state of the surrogate, the biphenyl being in a solid state at 55 °C, while it was liquid at the three other temperatures, and suggests that the tortuosity effect is not exerted in the same way in the case of the vacancy diffusion mechanism.

For temperatures below the biphenyl melting point (69.2 °C), the tortuosity effect is negligible compared to the loss of mobility of the surrogate. Additional measurements held at even lower temperatures would enable better characterization of such an effect. However, this shows that the physical state of the surrogate (liquid or solid) is a key factor in the quantification of the effect of nanoparticles on the decontamination of PE.

For temperatures higher than the biphenyl melting point, additional phenomena may occur, thus accounting for the tortuosity decrease with increasing temperature. Scientific research studies that focus on simpler clay/organic molecule systems detailed the temperature-dependent interactions that occur between the nano-sized platelets and the surrounding organic molecules [11,18,21,22,23,24,25]. Those affinities can be controlled by modifying the pristine mineral with quaternary ammonium surfactants, by increasing the specific surface of the mineral and rendering it hydrophobic and organophilic. This approach was historically developed to fight environmental pollutions by catching oily contaminating molecules [20,21] and was more recently found to benefit the elaboration of clay-nanocomposites by improving the interactions with macromolecules used as polymer matrices [11]. Reported results concerning affinities among clay/low molecular weight molecule systems provide useful information to understand the mechanisms governing the present clay/surrogate couple. Studies concerning contaminant removal by montmorillonite reported complex mechanisms, involving several mechanisms such as surface adsorption, partition, structural incorporation, and complex formation [23]. These sorption/desorption phenomena are driven by intermolecular forces (mainly Van der Waals forces and hydrogen bonding [24]) and desorption kinetics of volatile liquids from montmorillonite fit Arrhenius-type models [25]. As a consequence, increasing temperature accelerates these sorption/desorption phenomena and their contribution to the apparent tortuosity becomes negligible compared to the pure diffusion process. For the highest experimental temperature chosen in the present work (100 °C), the tortuosity is close to 1, thus indicating that the exchange phenomena occurring at the clay surface are negligible. However, working at 100 °C is not enough to circumvent the lowest apparent diffusivity of contaminants in nanocomposites: it takes 43% more time to decontaminate 95% of the biphenyl in nanocomposites than in pure PE (see Section 4.3).

Another interesting point provided by the decontamination studies concerns the role played by the structure of the quaternary alkylammonium surfactant—and more precisely the length of the hydrocarbon chains of the surfactant—which was reported to have a strong effect on the mechanism involved. In water, Smith et al. [25]. indicated that, for clays modified with surfactant whose hydrocarbon chains contained eight or fewer C atoms, the adsorption phenomenon was favored while, for chains with more than 14 C atoms, partitioning prevailed. In polymer-based nanocomposites, more efforts need to be made in order to validate such a hypothesis, especially for the surfactant used in the present work (dimethyl octadecyl ammonium) chosen for its good interactions with a PE matrix during the nanocomposite compounding. Even if it only represented a minor part of the global material composition (as PNC contains 5.9% mineral content, the quaternary ammonium corresponds to 5.9 × 38/62 = 3.6% of total nanocomposite weight), the constituent quaternary ammonium surfactants played a key role in the contaminant retention capacity reported here. Indeed, it modified the diffusion processes not only by increasing geometrical tortuosity through an improved clay platelet dispersion state, but also by strengthening the surrogate clay retention capacity.

## 5. Conclusions

While recycling technologies approved for food contact polyolefin-based materials are currently emerging, the introduction on the market of nanocomposite food packaging raises new questions about the impact of nanoparticles on flake-to-flake decontamination efficiency.

This study lays the groundwork for subsequent reflection on the possible upgrading of the decontamination process and future recommendations to be considered by safety authorities regarding the end-of-life treatment of nanocomposite packaging.

Decontamination of material results from both the diffusion of the pollutant (or surrogate) among the bulk of packaging material and its evaporation at the material surface. The incorporation of nanoclay into the polymer proved to influence the decontamination process by (i) slowing down the diffusion coefficient and (ii) limiting the evaporation of the surrogates at the material surface. The approach developed in this work made it possible to decouple the two phenomena.

The decrease in the diffusivity of the low molecular weight molecules induced by the presence of nanoclays is well established considering the geometrical tortuous path within the composite structure and the well-documented exchange phenomena that drive the clay/surrogate system. This effect proved to be additionally influenced by the physical state of the surrogate. The presence of NP within the polyethylene matrix seems to induce a retention of the diffusing contaminant leading to a reduction in its apparent volatility.

From a technical point of view and on the basis of the present result obtained on a unique surrogate, the specific behavior induced by the nano-sized mineral and its related quaternary ammonium surfactant should result in necessary new considerations regarding the conditions applied for the decontamination of polyethylene-based material. In particular, the quantified increase in the diffusion activation energy could help define new decontamination conditions (time and/or temperature) when nanocomposites are introduced in a physical recycling process.

In addition, the question of the behavior of nanoparticles during the recycling process remains open with regard to their possible destructuring and reorganization within the polymeric matrix. Answers will have to be provided on this issue before any recycling operation on nanocomposite packaging may be considered.

## Abbreviations

The following abbreviations are used in this manuscript:

PE	polyethylene
PNC	polymer nanocomposite
